# A dysglycaemic effect of statins in diabetes: relevance to clinical practice?

**DOI:** 10.1007/s00125-014-3409-3

**Published:** 2014-10-22

**Authors:** Daniel I. Swerdlow, Naveed Sattar

**Affiliations:** 1 Institute of Cardiovascular Science, University College London, 222 Euston Road, London, NW1 2DA UK; 2 BHF Glasgow Cardiovascular Research Centre, University of Glasgow, Glasgow, G12 8TA UK

**Keywords:** Cardiovascular disease, Dysglycaemia, HbA_1c_, Meta-analysis, Prevention, Statins, Type 2 diabetes

## Abstract

In this issue of the journal, Erqou and colleagues (DOI 10.1007/s00125-014-3374-x) report, in a systematic review and meta-analysis of randomised trials, a very modest (1.3 mmol/mol or 0.12%) albeit significant increase in HbA_1c_ in patients with diabetes treated with statins, compared with control. Here, we discuss the clinical relevance of the findings. Given the overwhelming benefit of statins on cardiovascular outcomes in diabetes, current guidelines recommending statins for primary prevention in type 2 diabetes should not change, and any effect on microvascular risk is likely to be minimal. Of course, all patients recommended for statin treatment, whether they have diabetes or not, should now be warned of a slight potential for dysglycaemia on starting statins, but at the same time they should be told that very modest lifestyle improvement will help offset this dysglycaemia risk. Finally, we remind colleagues that nearly all drugs have side effects and we should not be surprised by this statin–dysglycaemia effect, which can be easily managed.

The increased risk of new-onset type 2 diabetes associated with statin treatment is now well established. Large meta-analyses of randomised controlled trials (RCTs) of statins have demonstrated increased risk of developing type 2 diabetes when statins are compared with placebo or standard care [1], and when more intensive statin treatment is compared with less intensive [2]. Observational studies have also reported similar findings [3–5], although their design prevents inference of a causal role for statins. Nonetheless, one must remember that statins confer a substantial reduction in risk of cardiovascular disease (CVD) events in patients with and without established diabetes [6–8], so that the magnitude of CVD risk reduction for those eligible for statin treatment easily trumps any small increase in diabetes risk.

Nonetheless, there is now widespread interest in the nature of the relationship between statins and diabetes. Although RCT evidence suggests the effect is a consequence of statin treatment per se, observational studies have offered widely varying estimates of the magnitude of the association [3–5], and there has been uncertainty as regards the underlying biological mechanism [9]. A recent analysis of RCTs and genetic studies proposes a mechanistic link between statins and diabetes. Both statin treatment and variants in the gene encoding 3-hydroxy-3-methylglutaryl (HMG)-CoA reductase (*HMGCR*), the intended chemical target of statins, are associated with higher body weight and higher type 2 diabetes risk [10]. This consequence of statin treatment appears to be both on-target (i.e. mediated through HMG-CoA reductase inhibition) and, since higher body weight is known to be a causal factor in the development of diabetes [11], is likely to contribute at least in part to its diabetogenic effect.

Despite the growing body of evidence investigating the link between statins and new-onset diabetes, their effect on glycaemia in people with existing diabetes has attracted relatively limited consideration, with little published on the subject. In this issue of *Diabetologia*, Erqou and colleagues present evidence for a dysglycaemic effect of statins in individuals with diabetes [12], helping to fill this gap in the literature. The issue is an important one: diabetes is a well-established risk factor for CVD [13], and patients with diabetes are treated earlier and more intensively with statins than non-diabetic individuals are. Indeed, although the National Institute for Health and Care Excellence (NICE) has recently advised the reintroduction of risk scoring in type 2 diabetes, with statins recommended when the 10-year CVD risk is ≥10% [14], most countries adopt a 'fire and forget' approach, with all type 2 diabetic patients aged over 40 years offered statins [15], a position recently reiterated by the Joint British Societies (JBS)3 guidelines [16]. As statins are consequently prescribed to the overwhelming majority of patients with type 2 diabetes, investigating the influences of statin treatment on glycaemic control in these patients is merited.

The authors present a well-executed systematic review and meta-analysis of published summary-level results of statin trials that reported pre- and post-treatment HbA_1c_. The trials were largely conducted in patients with type 2 diabetes (although a small number included patients with type 1 diabetes or mixed samples), and the participants were characteristic of many patients being managed concurrently in the clinic for type 2 diabetes and CVD risk. The analyses included 9,696 individuals from nine trials, followed for an average of 3.6 years, and treated with a range of dosages of atorvastatin, pravastatin and simvastatin. Statin treatment led to a modest (0.12%; 1.3 mmol/mol) increase in HbA_1c_ when compared with the control, a finding that persisted in the type 2 diabetes-only subgroup, but was null in the smaller subgroup of type 1 diabetes and mixed populations. This study has a number of important strengths. First, it is the first substantial demonstration of a dysglycaemic effect of statin treatment among patients with existing diabetes. Second, the findings add weight to the growing body of evidence suggesting statin treatment is per se diabetogenic across a range of patients. This study is lent particular impact by its basis in randomised trials, enabling such causal inference as pharmacoepidemiological studies are unable to permit. Some weaknesses are also noteworthy. First, the number of trials with available relevant data is relatively small when compared with those used to quantify the effect of statins on risk of new-onset type 2 diabetes [10], and there was modest inter-study heterogeneity. Unpublished data are not included, and it is possible that these may harbour further helpful information; trial investigators should be motivated by this study to make such data accessible so that a more robust conclusion can be drawn. As the authors point out, data were unavailable on participants’ use of hypoglycaemic medication, and the range of statin drugs and dosages available was limited. It is possible that, as dysglycaemia progressed in statin-treated patients, their treating physicians may have intensified their hypoglycaemic therapy, leading to an attenuation of the observed statin–HbA_1c_ effect.

If one accepts the HbA_1c_ changes as real, the key question raised by this study, and other recent related findings, concerns its clinical implications, if any, for the care of patients with diabetes. CVD remains the chief cause of mortality and major morbidity among type 2 diabetic patients [13], and statins have been shown beyond doubt to reduce risk of CVD events in this population. Among type 2 diabetic patients, worsening dysglycaemia (indicated by higher HbA_1c_) only modestly raises risk of macrovascular disease such as myocardial infarction and ischaemic stroke [17, 18], being of greater relevance to microvasculature outcomes of nephropathy, retinopathy and peripheral neuropathy [17]. Furthermore, in patients with good medium- to long-term glycaemic control (HbA_1c_ <7% or 53 mmol/mol), there appears to be only a weak relationship between changes in HbA_1c_ and CVD risk [18]. The primary clinical value of statins lies in the prevention of macrovascular disease, an effect that is not meaningfully diminished by a small disturbance of glycaemic control (Fig. 1). There may, of course, be a very small effect on microvascular risk, but this question will be difficult to examine at an acceptable level of statistical power with the currently available trial data. The balance of benefit and risk falls, therefore, strongly in favour of continuing to prescribe statins for type 2 diabetic patients according to current guidelines. All such guidelines highlight the importance of lifestyle modification for the prevention of CVD and other diabetic complications. Evidence such as that presented by Erqou and colleagues for the adverse, but comparatively minor, effects of statins on glycaemia should encourage healthcare professionals to redouble their efforts to help their patients to improve their diets, engage in more physical activity and stop smoking. Even small and sustainable lifestyle changes can not only improve glycaemic control, but may also enhance patients’ quality of life in a win–win scenario. We must remember that few drugs lack adverse effects and that recent reductions in type 2 diabetes CVD mortality have likely largely resulted from improvements in cholesterol, blood pressure and smoking behaviours, as recently reviewed [19].

**Fig. 1:**
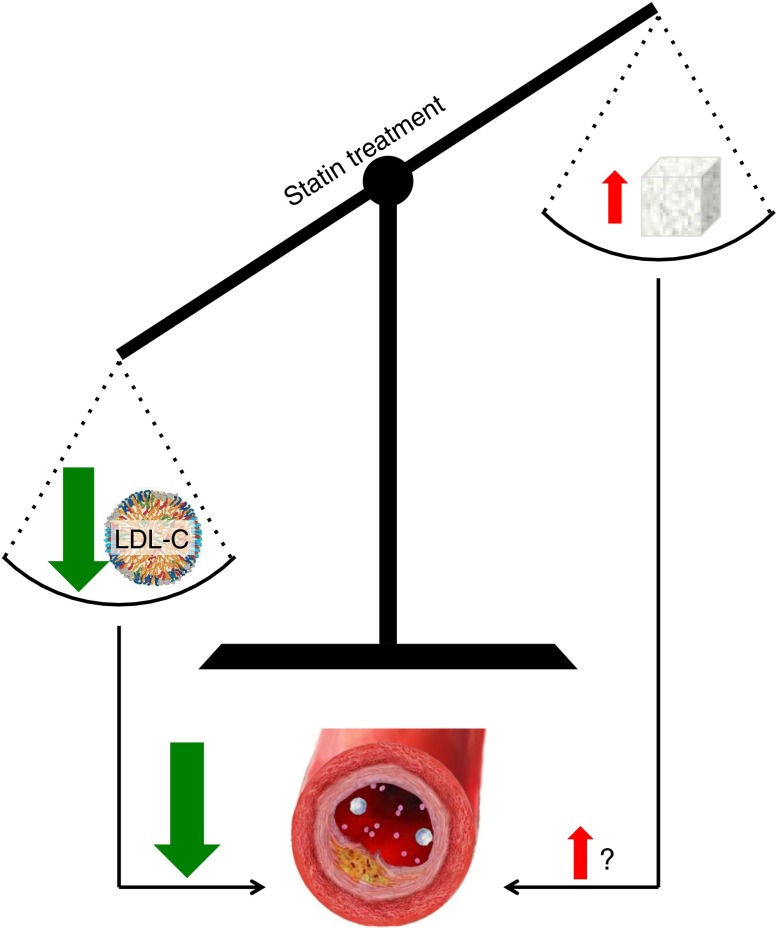
The beneficial effects of lipid-lowering by statins greatly outweighs the adverse effects of increased glycaemia for risk of macrovascular (atherosclerotic) disease. LDL-C, LDL-cholesterol

Finally, this study raises a further important issue regarding public and professional perceptions of statin drugs. Statins are not the only class of drug used in CVD prevention that raises plasma glucose concentration. Thiazide diuretics, for example, are commonly prescribed to patients with diabetes and have been shown to cause hyperglycaemia [20], but the spotlight of negative publicity appears to have fallen disproportionately on statins. This is likely because of the overwhelming frequency with which statins are prescribed, and the growing concern among patients and physicians about their other adverse effects. The bad press seems imbalanced, however, given both the considerable benefits for individual population health that these drugs confer and their excellent safety record, particularly when compared with other widely prescribed drugs such as aspirin. As mentioned above, no drug can be entirely free of adverse effects. However, the robustly demonstrated sizable benefits of statin treatment with minimal concomitant harm, and improved algorithms to handle statin intolerance [16] should remain foremost in the minds of the clinician and the patient when they are considering using a statin.

## Abbreviations

CVD: Cardiovascular disease
HMG: 3-Hydroxy-3-methylglutaryl
RCT: Randomised controlled trial
